# Gestational vitamin D and offspring fracture risk: do associations persist into mid adolescence?

**DOI:** 10.1038/s41430-024-01421-z

**Published:** 2024-03-01

**Authors:** Mia A. Percival, Kara B. Anderson, Julie A. Pasco, Sarah M. Hosking, Lana J. Williams, Kara L. Holloway-Kew, John D. Wark, Natalie K. Hyde

**Affiliations:** 1 Deakin University, IMPACT, The Institute for Mental and Physical Health and Clinical Translation, School of Medicine, Geelong, VIC 3220 Australia; 2 Barwon Health, Geelong, VIC 3220 Australia; 3 Department of Medicine-Western Health, The University of Melbourne, St Albans, VIC 3021 Australia; 4 Department of Epidemiology and Preventative Medicine, Monash University, Melbourne, VIC 3181 Australia; 5 Department of Medicine, Royal Melbourne Hospital, University of Melbourne, Parkville, VIC 3050 Australia; 6 Bone and Mineral Medicine, Royal Melbourne Hospital, Parkville, VIC 3050 Australia; 7 Department of Diabetes and Endocrinology, Royal Melbourne Hospital, Parkville, VIC 3050 Australia

**Keywords:** Epidemiology, Risk factors

## Abstract

**Background:**

Previous studies report that maternal vitamin D exposure during pregnancy is associated with offspring later-life bone health. A study in the Vitamin D in Pregnancy (VIP) cohort reported sexually dimorphic effects of maternal 25-hydroxyvitamin-D (25(OH)D) and offspring fracture profiles at 10 years of age. We, therefore, aimed to determine associations between maternal 25(OH)D status and offspring fracture risk at 16 years of age in this cohort.

**Methods:**

In total, 475 mother-child pairs were recruited to the VIP study in southeastern Australia. Maternal serum samples were obtained at recruitment (<16 weeks’ gestation) and/or 28–32 weeks’ gestation and analysed for 25(OH)D. Radiologically-confirmed incident fractures in children were ascertained from date of birth (2002–2004) until July 16, 2019. Cox proportional hazard models were used to determine associations between maternal 25(OH)D and childhood fracture risk, and final models included maternal age at recruitment, offspring sex, birth weight, gestation length and season of 25(OH)D sample.

**Results:**

Data were available for 400 children (mean age 16.1 years). There were 122 (30.5%) children who sustained at least one fracture. Higher maternal 25(OH)D (per 10 nmol/L) in early gestation was associated with a decreased fracture risk in boys (HR 0.87; 95% CI: 0.77, 0.99); the pattern was reversed in girls (HR 1.10; 95% CI 1.00, 1.22). At late gestation, higher maternal 25(OH)D was associated with an increased fracture risk in girls (HR 1.14; 95% CI: 1.04, 1.24).

**Conclusions:**

While our findings must be interpreted within the constraints of our limitations, we report that the contradictory risk profiles observed at early childhood in this cohort remain in adolescence.

## Introduction

Fractures in childhood are a costly and preventable injury accounting for almost 50% of all childhood injuries in Australia [1]. Increasing evidence suggests that maternal lifestyle and nutritional status during pregnancy are associated with offspring bone health, inclusive of fracture risk. For example, some studies have reported increased risk of fracture in offspring if the mother smoked cigarettes, followed a Western diet or consumed alcohol during pregnancy [2–4]. Vitamin D, in the form of 25(OH)D, is a precursor to 1α,25-dihydroxyvitamin D, a hormone important for bone formation, health and strength and the prevention of falls [5, 6]. Previous studies have reported contradictory associations between maternal vitamin D (25(OH)D) and offspring bone health, such as bone mineral density (BMD). Specifically, studies have reported that higher maternal 25(OH)D has been associated with both an increased and decreased BMD in their offspring at varying ages [5, 7–10].

An inverse association between maternal 25(OH)D status and offspring fracture risk until 18 years of age has been previously demonstrated; however, this study did not stratify by offspring sex [11]. We have previously reported that greater maternal 25(OH)D levels during early pregnancy (<16 weeks’ gestation) were associated with a lower fracture risk in boys at 10 years of age, whereas greater maternal 25(OH)D levels at mid to late gestation (28–32 weeks) were associated with an increased fracture risk in girls at 10 years of age [12]. Further follow-up data have since been collected allowing for the analysis of associations during adolescence. As fracture risk peaks in children between ages 11 and 12 years in girls, and 13 to 14 years in boys, [13, 14] we were interested to determine whether the previously observed patterns persist into this peak risk period. We, therefore, aimed to determine whether maternal 25(OH)D status during pregnancy was associated with offspring fracture risk from birth until 16 years of age and whether previously reported sexual dimorphism is persistent into adolescence.

## Methods

### Participants

Data were collected as part of the Vitamin D in Pregnancy (VIP) study, a longitudinal observational mother–child pair cohort study based in Geelong, southeastern Australia [15]. The methods for this study have been reported previously [12]. Briefly, 475 pregnant women were recruited from the antenatal clinic at the University Hospital Geelong (formerly the Geelong Hospital) between 2002 and 2004 and provided data, including blood samples, at recruitment (<16 weeks’ gestation) and 28–32 weeks’ gestation. Radiologically confirmed fractures were identified in the 402 offspring in whom data were provided for the study at birth.

### Data collection

#### Outcome: offspring fracture

Offspring fractures were identified for each child from their date of birth until July 16, 2019. Participants were linked to the Geelong Osteoporosis Fracture Grid as previously described, which collects data on radiologically-confirmed fractures in the region using an established protocol [16, 17]. In total, 400 (99.5%) of the original 402 children at birth had information available regarding their fracture history obtained from radiological reports. Two children were excluded as they were unable to be located in radiological records. All fracture sites and causes were included in this analysis.

#### Exposure: maternal vitamin D

Blood samples were obtained from mothers at recruitment (<16 weeks’ gestation) and 28–32 weeks’ gestation. Serum was stored at −70 °C and analysed for 25-hydroxyvitamin D (25(OH)D) by radioimmunoassay (Immunodiagnostic Systems, Tyne and Wear, UK). The assay used for these samples reports a 75% reactivity with 25(OH)D_2_ and 100% with 25(OH)D_3_. The coefficient of variation was 10.2% at 30 nmol/L and 10.1% at 100 nmol/L. Blood sample testing was performed at the Royal Children’s Hospital, Melbourne. As previously described, a variable for the season of serum sample was calculated using the day of sample collection [18].

#### Other measurements

At recruitment and 28–32 weeks’ gestation, maternal height was measured using a Harpenden stadiometer (±0.1 cm) and weight using electronic scales (±0.1 kg). Self-reported smoking status was collected.

Offspring sex, birth weight (±0.1 g), length (±0.1 mm), knee-heel length (±0.1 mm) and gestation length (weeks) were recorded following birth by trained professionals.

Area-based socioeconomic status was determined by matching the residential address at recruitment to the Australian Bureau of Statistics’ Index for Relative Disadvantage deciles using data from the 2001 census and then collapsed into quintiles [19].

### Statistical analysis

Demographic data were compared between children who did and did not sustain a fracture between birth and the end of follow up (mean 16.1 years). Mann–Whitney *U* tests were used for non-parametric, continuous variables, and chi-squared tests for categorical variables. Associations between maternal 25(OH)D status and offspring fracture risk were analysed using Cox proportional hazard models with age as the time scale. Children were at risk for a fracture from the time of birth until the age of first fracture or the censor date (July 16, 2019), whichever occurred first. Maternal 25(OH)D values were entered into models as continuous and categorical variables (cut-points of 28 nmol/L, 50 nmol/L and 75 nmol/L). These categorical cut points were used as <28 nmol/L was used in original analysis in this cohort, 50nmol/L is the recommended vitamin D level by the Australian Position Statement and a vitamin D level of 75 nmol/L or more has been suggested as an appropriate cut-off for optimal bone health in a prior study [20–22]. A sex interaction term between each maternal 25(OH)D variable (continuous/categorical at each time point) and offspring sex was tested in the corresponding model. A conservative approach was taken, whereby significance was set at *p* = 0.1 when considering the interaction term. If the term was significant for the analysis, the results were stratified by sex. Model 1 was unadjusted. Model 2 included Model 1 and the season of 25(OH)D sample and offspring sex. Model 3 included all variables presented in Model 2 with the addition of maternal age at recruitment, birthweight and gestation length. All analyses were performed using Stata (version SE 17, StataCorp LLC, College Station, TX). Significance was set at *p* = 0.05.

### Ethics

This study was approved by Barwon Health Human Research Ethics Committee (01/43). All mothers and/or guardians of children in this study provided consent on behalf of themselves and their children.

## Results

### Demographics

**Fig. 1:**
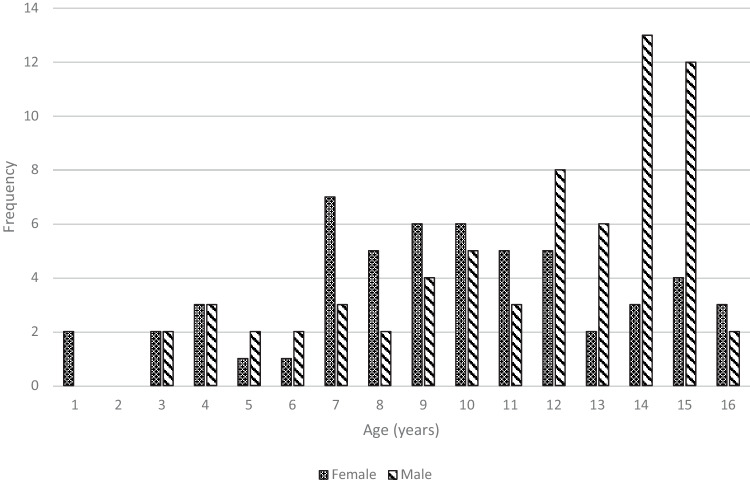
Age of first fracture in boys and girls.

Overall, 122 of the 400 (30.5%) children sustained at least one recorded fracture during follow-up, 67 (54.9%) were boys and 55 (45.1%) were girls. Maternal and offspring characteristics of children who did and did not sustain a fracture during the study period were similar (Table 1, Fig. 1). The median age of fracture was 12.5 years (IQR 9.3–14.4) in boys and 9.6 years (IQR 7.1–12.4) in girls. There were 181 fractures during the study period, the fracture sites and causes are summarised in Table 2. Of those experiencing a fracture, 82 (67.2%) children had one fracture, 26 (21.3%) had two fractures, nine (7.4%) had three fractures and five (4.1%) had four fractures during the study period.

**Table 1:** Characteristics of mothers and offspring according to offspring fractures.

Characteristics	Fracture		*p*-value
	No (*n* = 278)	Yes (*n* = 122)	
Mothers (recruitment<16 weeks’ gestation)			
Socioeconomic status (SES)			0.45
1 (most disadvantaged)	63 (22.8)	36 (29.5)	
2	66 (23.9)	33 (27.1)	
3	64 (23.2)	25 (20.5)	
4	40 (14.5)	14 (11.5)	
5 (least disadvantaged)	43 (15.6)	14 (11.5)	
Smoker *n* (%)	50 (18.3)	21 (17.5)	0.86
Weight (kg)	69.0 (60.8–79.1)	69.3 (61.0–84.0)	0.48
Height (cm)	165.5 (162.1–169.8)	163.8 (160.5–168.5)	0.23
Age at recruitment (yr)	29.1 (25.7–32.5)	29.2 (25.6–32.4)	0.94
25(OH)D concentration (nmol/L)	54.8 (41.6–69.9)	55.0 (40.3–67.3)	0.79
Mothers (28–32 weeks’ gestation)			
Weight (kg)	77.0 (68.4–87.0)	76.5 (68.7–91.0)	0.47
25(OH)D concentration (nmol/L)	55.5 (40.8–80.9)	56.9 (44.0–79.5)	0.59
Offspring			
Gestation length (weeks)	40.0 (39.0–41.0)	40.0 (38.0–40.0)	0.57
Sex			
Male	128 (46.0)	67 (54.9)	0.10
Birth weight (kg)	3.5 (3.2–3.9)	3.6 (3.3–3.9)	0.30
Birth length (cm)	50.5 (48.6–52.0)	50.4 (48.9–51.8)	0.66
Knee Heel Length (mm)	86.4 (80.8–91.4)	85.9 (80.6–90.0)	0.40
Age at end of study period (yr)	16.1 (15.7–16.4)	16.1 (15.8–16.5)	0.54

Presented as median (interquartile range) or *n* (%).

**Table 2:** Characteristics of offspring fractures.

Fracture characteristics^a^	*n* (%)
Fracture Site	
Radius or ulna	82 (45.3)
Finger	27 (14.9)
Humerus	15 (8.3)
Foot	13 (7.2)
Tibia or Fibula	11 (6.7)
Toe	11 (6.7)
Clavicle	10 (5.5)
Hand	7 (3.9)
Other^b^	5 (2.8)
Fracture Cause	
Accidental falls from one level to another or on the same level	67 (37.0)
Struck by an object accidentally	10 (5.5)
Caught between objects accidentally	5 (2.8)
Overexertion or strenuous movement	4 (2.2)
Falling from a bike/animal being ridden	4 (2.2)
Accidents caused by cutting and piercing objects	1 (0.6)
Accidents involving a motor vehicle	1 (0.6)
Unreported	89 (49.2)

^a^Participants may have had more than one fracture during the study period. Percentages (%) are out of 181 fractures.

^b^Includes face, spine, femur and patella.

In total, 382 (95.0%) and 376 (93.5%) of the 402 mother-child pairs had complete data and were included in analyses at recruitment and 28–32 weeks, respectively.

### Maternal vitamin D at recruitment

Maternal 25(OH)D status at recruitment was not associated with offspring fracture risk in the unadjusted model (Table 3). A sex interaction term was significant; thus, analyses were stratified by offspring sex. An association between vitamin D status and offspring fracture risk became significant for both boys and girls in the sex-stratified model, after adjusting for season of 25(OH)D sampling (Model 2). This association remained significant after final adjustment for confounders (Model 3) in boys but was attenuated in girls.

**Table 3:** Hazard ratios of maternal vitamin D (continuous and categorical) in models predicting offspring fracture.

	Continuous 25(OH)D		Above or below 28 nmol/L		Above or below 50 nmol/L		Above or below 75 nmol/L	
	HR (95% CI)	*p*	HR (95% CI)	*p*	HR (95% CI)	*p*	HR (95% CI)	*p*
Recruitment								
Sex interaction term		**0.011**		0.40		**0.036**		**0.055**
Model 1								
All	-	-	1.04 (0.49–2.23)	0.92	-	-	-	-
Female	1.10 (0.99–1.22)	0.072	1.60 (0.39–6.57)	0.51	1.67 (0.92–3.03)	0.089	1.59 (0.89–2.84)	0.12
Male	0.90 (0.79–1.01)	0.069	0.80 (0.32–1.98)	0.63	0.74 (0.46–1.20)	0.23	0.71 (0.39–1.30)	0.27
Model 2								
All	**-**		1.03 (0.48–2.22)	0.94	-	-	-	-
Female	**1.10 (1.00–1.23)**	**0.042**	1.66 (0.40–6.87)	0.48	1.77 (0.96–3.26)	0.069	1.55 (0.79–3.06)	0.20
Male	**0.87 (0.77–0.99)**	**0.030**	0.70 (0.28–1.78)	0.46	0.67 (0.40–1.10)	0.12	0.61 (0.32–1.19)	0.15
Model 3								
All	-	-	1.00 (0.46–2.17)	1.00	**-**	**-**	-	-
Female	1.10 (1.00–1.22)	0.053	1.40 (0.34–5.84)	0.65	**1.96 (1.03–3.71)**	**0.040**	1.55 (0.79–3.05)	0.21
Male	**0.87 (0.77–0.99)**	**0.033**	0.72 (0.28–1.83)	0.49	0.65 (0.39–1.09)	0.10	0.56 (0.30–1.17)	0.13
28–32 weeks gestation								
Sex interaction term		**0.034**		0.21		0.17		**0.009**
Model 1								
All	**-**	**-**	1.03 (0.52–2.03)	0.93	1.30 (0.89–1.90)	0.18	-	-
Female	**1.09 (1.01–1.19)**	**0.035**	0.67 (0.27–1.69)	0.40	1.67 (0.92–3.03)	0.089	1.64 (0.96–2.78)	0.069
Male	0.96 (0.87–1.05)	0.37	1.60 (0.58–4.39)	0.36	0.74 (0.46–1.20)	0.23	0.56 (0.31–1.03)	0.061
Model 2								
All	**-**	**-**	1.16 (0.58–2.30)	0.68	1.48 (0.99–2.20)	0.056	**-**	**-**
Female	**1.11 (1.03–1.22)**	**0.009**	0.69 (0.27–1.76)	0.44	1.53 (0.83–2.84)	0.17	**1.80 (1.00–3.24)**	**0.050**
Male	0.97 (0.87–1.07)	0.51	1.82 (0.66–5.05)	0.25	0.69 (0.41–1.16)	0.16	0.67 (0.34–1.31)	0.24
Model 3								
All	**-**	**-**	1.31 (0.63–2.71)	0.48	**1.56 (1.04–2.36)**	**0.032**	**-**	**-**
Female	**1.14 (1.04–1.24)**	**0.004**	0.89 (0.31–2.55)	0.83	1.62 (0.85–3.08)	0.14	**1.94 (1.06–3.57)**	**0.032**
Male	0.96 (0.87–1.06)	0.43	1.83 (0.66–5.09)	0.25	0.64 (0.38–1.09)	0.10	0.63 (0.31–1.27)	0.20

Model 1- unadjusted, Model 2- Model 1 + offspring sex and season of 25(OH)D sample, Model 3- Model 2 + birthweight, gestation length and maternal age at recruitment. Bold values denote a statistical significance of *p* < 0.1 for sex interaction terms and *p* < 0.05 for Cox proportional hazard models.

In total, 23 (6.0%), 154 (40.3%) and 308 (80.6%) mothers had 25(OH)D levels below 28 nmol/L, 50 nmol/L and 75 nmol/L at recruitment, respectively (Supplementary Table 1).

Maternal 25(OH)D status at recruitment was not associated with offspring fracture risk when analysed as a categorical variable with the 28 nmol/L or 75 nmol/L cut points (Table 3). However, using the 50 nmol/L cut point, the final adjusted model was significant in girls only. In this model, girls with mothers with a 25(OH)D level above 50 nmol/L at recruitment were nearly twice as likely to fracture a bone during the study period as girls with mothers with 25(OH)D below 50nmol/L (HR 1.96; 95% CI: 1.03, 3.71; *p* = 0.040).

### Maternal vitamin D at 28–32 weeks’ gestation

Maternal 25(OH)D status at 28–32 weeks’ gestation was associated with offspring fracture risk in girls in the unadjusted model (Model 1, see Table 3). This association remained significant after final adjustment for confounders (Model 3). For every 10 nmol/L increase in maternal 25(OH)D at 28–32 weeks’ gestation, girls were 14% more likely to fracture a bone in the study period (HR 1.14; 95% CI: 1.04,1.24; *p* = 0.004). There was no association observed for boys at this time point.

In total, 29 (7.7%), 148 (39.4%) and 270 (71.8%) mothers had 25(OH)D levels below 28 nmol/L, 50 nmol/L and 75 nmol/L at 28–32 weeks’ gestation, respectively (Supplementary Table 1).

Maternal 25(OH)D status at 28–32 weeks’ gestation was not associated with offspring fracture risk when analysed as a categorical variable with the 28 nmol/L cut point (Table 3). The final model (Model 3) was significant at the 50 nmol/L cut point, with analysis pooled as the sex interaction term was not significant when considering this cut point (*p* = 0.174). Thus, children of mothers with 25(OH)D levels above 50 nmol/L at 28–32 weeks’ gestation were 56.4% more likely to fracture a bone in the study period than children of mothers with 25(OH)D levels below 50nmol/L (HR 1.56; 95% CI: 1.04, 2.36; *p* = 0.032). Using the 75 nmol/L cut point, the final adjusted model (Model 3) was not significant in boys (HR 0.63; 95% CI: 0.31, 1.27; *p* = 0.20). However, it was associated with an increased fracture risk in girls, whereby girls of mothers with a 25(OH)D level above 75nmol/L were 94.5% more likely to fracture a bone in the study period than those of mothers with a 25(OH)D level below 75 nmol/L (*p* = 0.032).

## Discussion

Here, we report that higher maternal 25(OH)D levels during early gestation were associated with a lower fracture risk in boys, but not girls at 16 years of age. In comparison, higher maternal 25(OH)D at late gestation was associated with an increased fracture risk in girls, but not boys.

We have previously demonstrated that maternal 25(OH)D status during early and late pregnancy was associated with offspring fracture risk at 10 years [12]. Of note, patterns observed in this study, with fracture ascertainment extended to 16 years of age, are the same as previously reported. There was a 13.0% decreased fracture risk in boys who had been exposed to higher maternal 25(OH)D at recruitment and a 13.6% increase in fracture risk in girls who had been exposed to higher maternal 25(OH)D at 28–32 weeks’ gestation in this study. A recent randomised controlled trial of vitamin D supplementation in pregnancy and offspring bone health reported a lower fracture risk in the high dose supplementation group in early childhood, which aligns with our findings in early pregnancy with boys; however, this study did not stratify the results by sex and therefore it cannot be determined if there are differential risk profiles between the sexes, as in our study [23, 24].

Interestingly in this study, categorical 25(OH)D at 28–32 weeks’ gestation was associated with a 56.4% increase in fractures in children with mothers with 25(OH)D greater than 50 nmol/ L (*p* for interaction 0.174); however, this appears to be driven by the girls, and again an almost doubling in fracture risk in girls with mothers with 25(OH)D levels greater than 75 nmol/L. A U-shaped curve has been reported previously in studies of adults supplemented with vitamin D and fracture risk, suggesting that both very low and high levels are detrimental for fracture risk [25–27]. A study in mice also reported that mothers with elevated 25(OH)D levels during pregnancy had offspring with defects in calcium incorporation into bones, resulting in lower bone mass and reduced mineralisation, but this has not been reported in human studies [28]. The authors discussed potential reasons for this observation, noting that 25(OH)D crosses the placenta and, in the case where 25(OH)D levels are significantly elevated, leads to reduced formation of the inorganic phosphate necessary for mineralisation, as well as an increase in inhibitors of bone mineralisation. It is plausible that high intake of vitamin D, and high levels of 25(OH)D, may lead to the production of vitamin D metabolites that are partial agonists, or antagonists, at the vitamin D receptor and therefore, may have a detrimental influence on bone signalling pathways. However, this needs further investigation.

The reason for these associations being driven by girls in our study remains unclear, however, as alluded to in the earlier paper, sexually dimorphic effects have been reported in other Developmental Origins of Health and Disease studies and may be associated with sex specific differences in the action of the placenta [29]. In particular, there is evidence of sex-specific differences in placental genes important for vitamin D metabolism [30]. Workalemahu et al. reported that higher maternal vitamin D during pregnancy was associated with a higher birthweight in male offspring and lower birth weight in female offspring. With previous studies linking lower birthweight to lower bone mineral content and density throughout the lifespan, and lower bone mineral content and density being linked to increased risk of fractures, it is reasonable to suggest that the pathways contributing to birthweight may also be contributing to fracture risk [31–33]. However, we did adjust for birthweight in the models and the associations remained. Nevertheless, vitamin D may be acting through another placental pathway and may be contributing to the sexual dimorphism observed in this study.

Furthermore, there may be action through sex hormones. Some studies report that higher levels of vitamin D may reduce oestrogen and increase testosterone expression in the body [34–37]. Therefore, there may be action of vitamin D that causes reductions in the expression of foetal oestrogen in female offspring and increases in testosterone expression in male offspring. Lower levels of oestrogen and testosterone have been implicated in decreases in bone mineral density and content, and increases in fracture risk, in post-menopausal women and older men [38–42]. While this has not been studied in pregnant women and their offspring, this may explain the findings of our study with increases in female offspring fracture risk and decreases in male offspring fracture risk with increasing maternal vitamin D.

These findings are not without limitations. It is possible that some fractures were missed due to being managed outside the study region. If the fracture occurred outside the region or the family had moved, or the fracture was managed at a smaller radiological centre, these would not be captured in this study. However, it is likely that if a fracture occurred outside the region a follow-up x-ray would have been completed within the region and picked up in our data collection. It is also well documented that vitamin D levels fluctuate with the season, thus associations could be explained by other factors with similar seasonal variations. However, we did adjust for seasonal variation in models. Furthermore, fracture risk is dependent on many other factors that we were unable to assess in this study. In particular, the child’s participation in sport, which may increase fracture risk, and their bone characteristics and sex hormones, which may affect bone growth, strength, structure and metabolism, and therefore may predispose a child to higher or lower fracture risk. A major strength of this study is the prospective ascertainment of fractures from radiology reports and the objective measures of maternal 25(OH)D at early and late gestation.

To our knowledge, this is the first study to report the relationship between maternal 25(OH)D status at early and late gestation and offspring fracture risk after the typical age of the pubertal growth spurt [43]. While our findings must be interpreted within the constraints of our limitations, we report that the contradictory risk profiles seen at early childhood in this cohort remain in adolescence. These findings should be followed up in larger cohorts to ascertain whether associations are indeed true associations and whether they are mediated by factors such as risk-taking behaviour, changes in bone density, the offspring’s own 25(OH)D status or sex-specific differences in vitamin D action of which we were unable to test for in this study.

### Supplementary information

Supplementary Table 1

## Data Availability

Data will be made available upon reasonable requests and subject to ethical approval.
