# Supplementary material for: Gestational vitamin D and offspring fracture risk: do associations persist into mid adolescence?
Source: Eur J Clin Nutr. 2024 Mar 1;78(6):515–20. doi: 10.1038/s41430-024-01421-z (PMC11182745; doi:10.1038/s41430-024-01421-z)
Supplement: Supplementary file 1 — Supplementary Table 1 [file 41430_2024_1421_MOESM1_ESM.docx]

Supplementary Table 1: The number of mothers and number of children with a fracture per categorical cut point

|  | **Number of mothers** | **Number of children with a fracture** |
| --- | --- | --- |
| **Recruitment** | | |
| ≤28nmol/L | 23 | 7 |
| >28 nmol/L | 359 | 107 |
| ≤50nmol/L | 154 | 46 |
| >50nmol/L | 228 | 68 |
| ≤75nmol/L | 308 | 93 |
| >75nmol/L | 74 | 21 |
| **28-32 weeks gestation** | | |
| ≤28nmol/L | 29 | 9 |
| >28 nmol/L | 347 | 107 |
| ≤50nmol/L | 148 | 40 |
| >50nmol/L | 228 | 76 |
| ≤75nmol/L | 270 | 84 |
| >75nmol/L | 106 | 32 |
